# Anti-fungal activity of moso bamboo (*Phyllostachys pubescens*) leaf extract and its development into a botanical fungicide to control pepper phytophthora blight

**DOI:** 10.1038/s41598-021-83598-y

**Published:** 2021-02-18

**Authors:** Min Liao, Xuexiang Ren, Quan Gao, Niuniu Liu, Feng Tang, Ge Wang, Haiqun Cao

**Affiliations:** 1 Anhui Province Key Laboratory of Crop Integrated Pest Management, School of Plant Protection, Anhui Agricultural University, Hefei, China; 2 Anhui Province Engineering Laboratory for Green Pesticide Development and Application, School of Plant Protection, Anhui Agricultural University, Hefei, China; 3 Institute of Plant Protection and Agro-Products Safety, Anhui Academy of Agricultural Sciences, Hefei, China; 4 School of Resource and Environment, Anhui Agricultural University, Hefei, China; 5 State Forestry Administration Key Open Laboratory, International Centre for Bamboo and Rattan, Beijing, China; 6 Key Laboratory of Bamboo and Rattan Science and Technology of the State Forestry Administration, Department of Bio-Materials, International Centre for Bamboo and Rattan, Beijing, China

**Keywords:** Secondary metabolism, Analytical chemistry

## Abstract

Moso bamboo (*Phyllostachys pubescens*, Gramineae) is a well-known medicinal and edible plant found in China with various bioactivities, but few systematic studies address the utilization of its anti-fungal activity. The extract of moso bamboo leaf showed good anti-fungal activity to *Phytophthora capsici*, *Fusarium graminearum*, *Valsa mali* Miyabe et Yamada, *Botryosphaeria dothidea*, *Venturia nashicola*, and *Botrytis cinerea* Pers, with inhibitory rate of 100.00%, 75.12%, 60.66%, 57.24%, 44.62%, and 30.16%, respectively. Anti-fungal activity was different by the difference of samples picking time and location. The extract showed good synergistic effects with carbendazim at the ratios of 9:1 and 15:1 (extract : carbendazim), and the co-toxicity coefficients were 124.4 and 139.95. Compound **2** was isolated and identified as the main active component, with the EC_50_ value of 11.02 mg L^−1^. Then, the extract was formulated as a 10% emulsion in water, which was stable and had no acute toxic effects. Moreover, a field trial about this formulation was assayed to control pepper phytophthora blight, with the control effect of 85.60%. These data provided a better understanding of the anti-fungal activity and relevant active component of moso bamboo leaf extract. Taken together, our findings illustrated that bamboo leaf extract could be developed and utilized as a botanical fungicide or fungicide adjuvant.

## Introduction

Plant disease is a major factor constraining many aspects of the sustainable development of agriculture. The occurrence of plant diseases reduces crops including vegetables and fruits production, and affects quality directly^1,2^. Synthetic pesticides have made an important contribution to global agricultural production, and without pesticides, the yields of crops will be reduced by more than 30%^3^. However, the widespread use of synthetic pesticides has resulted in adverse effects for both wildlife and humans, in addition, the influence of environmental and agricultural product pollution, ecological imbalance, food safety, including residue, resistance and resurgence are also serious problems^4,5^. Other plant diseases mentioned in this study seriously affect the yield and quality of fruits and vegetables. For example, *Phytophthora capsici* can cause diseases in many important vegetable crops, such as pepper, eggplant, tomato, and cucumber, which is a kind of devastating disease^6,7^. The long-term use of traditional synthetic fungicides to control the disease leads to increasing of resistance, pesticide residue and environmental pollution^8^. Discovery of new plant resources with anti-fungal activity has great significance^9^. It has become a popular research topic to find and develop new types of biological pesticides from biological resources that are environmentally friendly and safe and that leave low amounts of residue in the environment^10^.

The active ingredients of botanical pesticides are diversified, making it difficult to develop drug resistance, and they are safer for beneficial organisms^11,12^. Approximately 4000 plants have preventive effect on pests and diseases in crops, and plants with antimicrobial activity account for nearly 1/3 of all plants^13^. Botanical fungicide refers to an agent that controls plant diseases by using certain parts of a plant with fungicidal activity or by extracting an active ingredient from one of these plants, with a wide range of sources and are present in various plant extracts. Therefore, studies about new botanical pesticides and related dosage forms are important to the development of green agriculture.

The processing of pesticide preparations is an important part of the pesticide industry and the last step in the commercialization of pesticides^14^. Current environmental protection regulations have put forward high requirements for pesticide formulations^15^. In recent years, emulsifiable concentrates have been restricted or banned by developed countries due to their high organic solvent content, which was replaced by high-performance, environmentally friendly water-based pesticide formulations^16^. The industrialization of new formulations of water-based pesticides is increasing at a rate of 10–30% every year, such as emulsion in water (EW), suspension concentrate (SC), water dispersible granule (WDG) and micro-emulsion (ME)^17,18^. Aqueous preparations can conserve many organic solvents and greatly reduce environmental pollution and human harm^19^. Moreover, natural products can delay the development of resistance in harmful organisms because of their complexity and other characteristics, reduce the amount of synthetic pesticides used, and improve the safety of pesticide application^20,21^. Recent studies have shown that a variety of natural products can be used as auxiliaries for synthetic fungicides, such as bamboo vinegar, alliospiroside, and cashew nut shell oil^22–24^.

Moso bamboo (*Phyllostachys pubescens*, Gramineae) (*syn. edulis*) is one of the main bamboo species and an important forest resource in China^25^. Because moso bamboo is a medicinal and edible plant found in China, the study of its biological activity and chemical compositions is an important topic that requires more in-depth research^26,27^. The extracts of bamboo have a variety of physiological functions, such as insecticidal activity, anti-ageing, and antitumour^28,29^. In addition, multiple bio-active substances have been found in bamboo, such as flavonoids, amino acids, polysaccharides, and alkaloids^30,31^. The extract of bamboo was reported to have anti-fungal activity^32^, however, studies on the main active components and application of moso bamboo extract are scarce.

In this study, the anti-fungal activity of bamboo leaf extract against the phytopathogens *P. capsici*, *Fusarium graminearum*, *Valsa mali* Miyabe et Yamada, *Botryosphaeria dothidea*, *Venturia nashicola* and *Botrytis cinerea* Pers was assayed. The influence of bamboo leaf collected time and location to anti-fungal activity was compared. Then, the main active component of anti-fungal activity in moso bamboo extract was isolated and identified, and the activity was validated. In addition, synergistic effect of the extract on carbendazim was investigated. Finally, field test of the 10% EW of BE control pepper phytophthora blight was performed according to national standards.

## Results

### Extract of moso bamboo leaf inhibits the growth of *P. capsici*, *F. graminearum*, *V. mali*, *B. dothidea*, *V. nashicola* and *B. cinerea* in vitro

**Figure 1:**
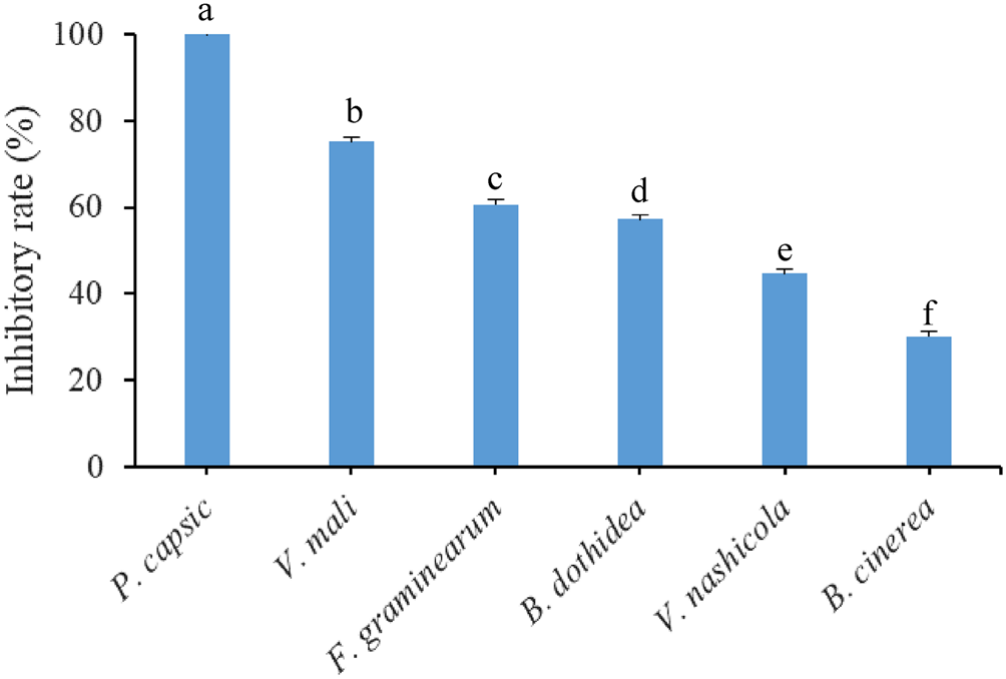
Anti-fungal activity of moso bamboo leaf extract. The treatment concentration was 5.0 g L^−1^, average data are presented and error bars represent the standard error of the mean (n = 6). Different letters indicate significant differences according to Tukey HSD test comparisons (*p* < 0.05).

**Figure 2:**
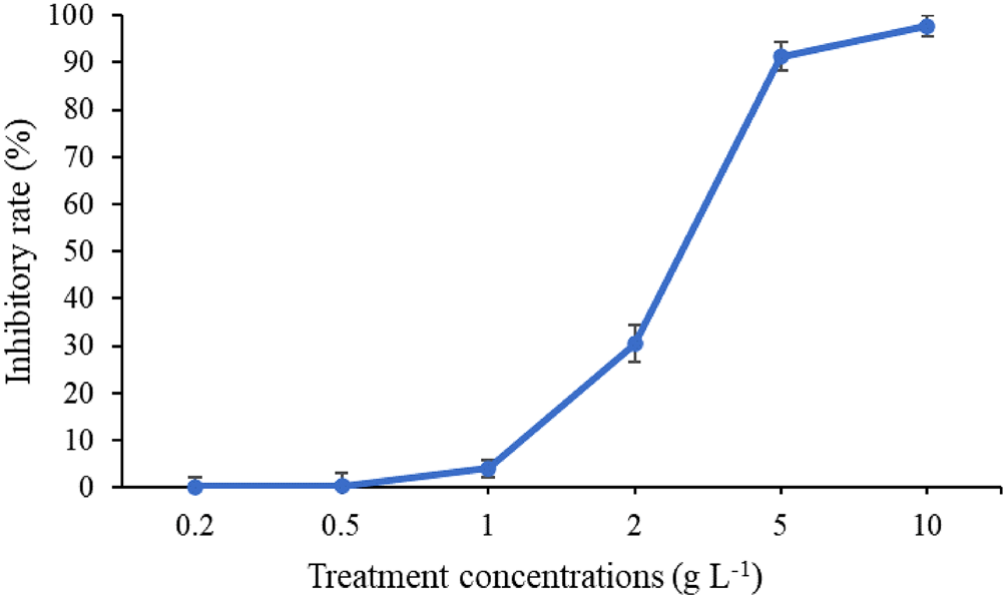
Moso bamboo leaf extract inhibits the growth of the phytopathogen *P. capsici*.

The extract of moso bamboo leaf showed good anti-fungal activity against the tested fungi species (Fig. 1). When the concentration was 5.0 g L^−1^, the inhibitory rates to *P. capsici*, *V. mali* and *F. graminearum* were 100.00 ± 0.1%, 75.12 ± 0.92% and 60.66 ± 3.35%, respectively. In addition, the EC_50_ value of BE against *P. capsici* was 2.52 g L^−1^ (Fig. 2).

**Figure 3:**
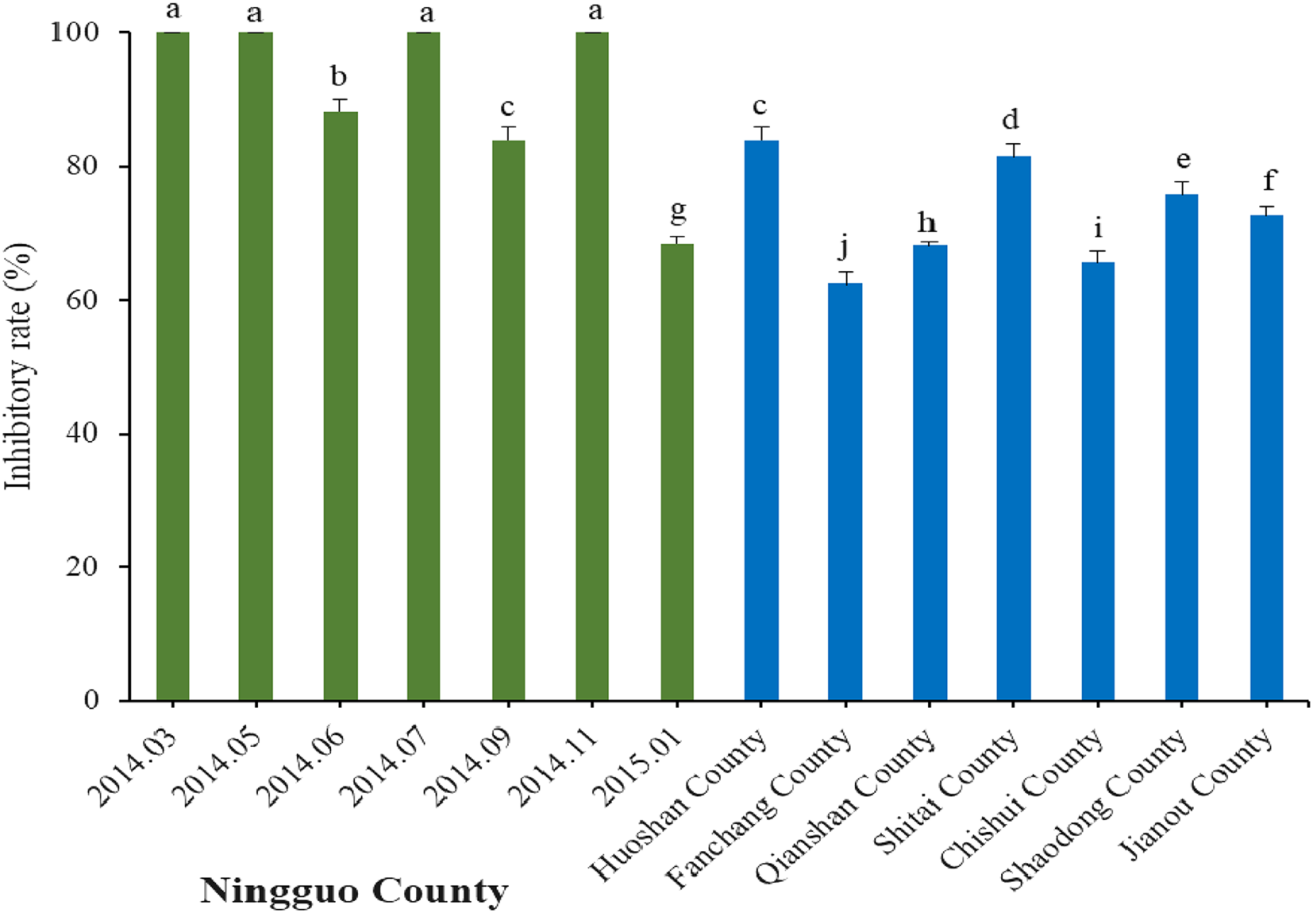
Anti-fungal activity of bamboo leaf extract against *P. capsici*. Note that the bamboo leaves were collected from different locations and at different times in Ningguo County. Average data are presented and error bars represent the standard error of the mean (n = 6). Different letters indicate significant differences according to Tukey HSD test comparisons (*p* < 0.05).

Then, the influence of bamboo leaf collected time and location to anti-fungal activities against *P. capsici* were investigated with same hyphal growth rate method. The results indicated that the BE collected in Ningguo, Huoshan and Shitai Counties showed better anti-fungal activity against *P. capsici* than the BE collected in the other counties (Fig. 3). When the concentration was 5.0 g L^−1^, the average inhibitory rates were 92.31 ± 1%, 83.98 ± 1.8% and 81.46 ± 1.92% in 48 h, respectively. Next, the BE from Shaodong County and Jianou County had inhibitory rates of 75.69 ± 1.97% and 72.66 ± 1.13%, respectively. The inhibitory rate was 100.00% with samples collected in March, May, July, and November 2014.

### BE has a synergistic effect with carbendazim

When BE was combined with carbendazim at ratios of 5:1, 7:1, 9:1, 15:1, 49:1 and 99:1, the EC_50_ values were 2.37 × 10^−2^, 2.56 × 10^−2^, 2.25 × 10^−2^, 3.20 × 10^−2^, 23.20 × 10^−2^, and 191.30 × 10^−2^ mg L^−1^, respectively. The co-toxicity coefficients (CTCs) were 70.88, 87.50, 124.40, 139.95, 26.64, and 14.63, respectively. These results indicated that BE showed a synergistic effect when the ratios were 9:1 and 15:1 (Table 1), revealing that the observed synergism was significant.

**Table 1:** The synergistic effects of BE with carbendazim.

Treatment BE : carbendazim	Toxicity regression equation	EC_50_ (mg L^−1^)	Relative coefficient	CTC	95% Confidence intervals (mg L^−1^)
5:1	y = 1.6555x + 4.3786	2.37 × 10^–2^	0.9893	70.88	2.08 × 10^–2^–2.74 × 10^–2^
7:1	y = 2.0388x + 4.1671	2.56 × 10^–2^	0.9928	87.50	2.29 × 10^–2^–2.85 × 10^–2^
9:1	y = 1.1738x + 4.3865	2.25 × 10^–2^	0.9846	124.44	1.89 × 10^–2^–2.66 × 10^–2^
15:1	y = 1.2661x + 4.3554	3.20 × 10^–2^	0.9948	139.95	2.88 × 10^–2^–3.56 × 10^–2^
49:1	y = 2.2446x + 1.1381	23.20 × 10^–2^	0.9894	26.64	47.79 × 10^–2^–57.85 × 10^–2^
99:1	y = 1.3652x + 1.8851	191.30 × 10^–2^	0.9865	14.63	166.6 × 10^–2^–223.20 × 10^–2^

### Isolation and structural elucidation of main active compound

**Figure 4:**
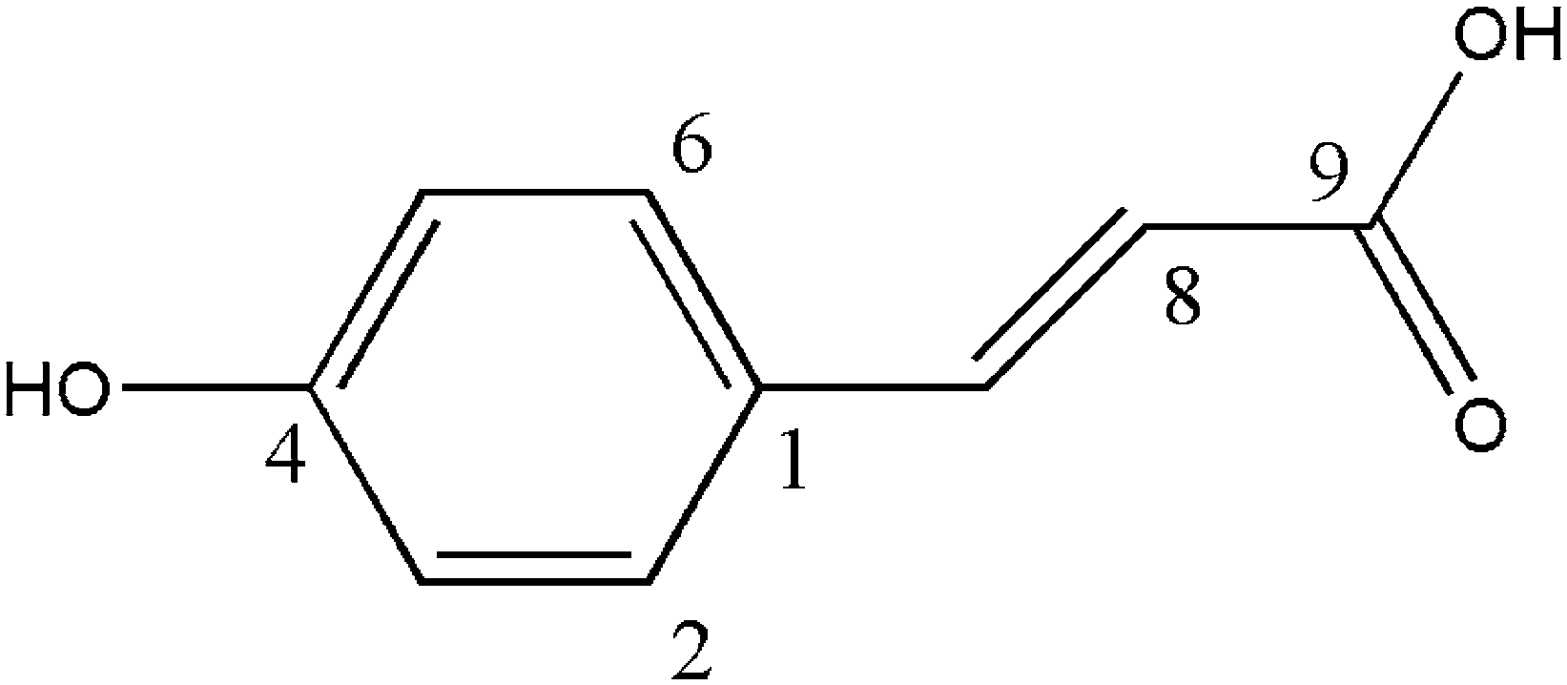
The structure of compound **2**.

Compound **2** was obtained as a white amorphous powder. UV spectrum exhibited absorption maxima at 225.0 and 309.3 nm. The molecular formula was established as C_9_H_8_O_3_ according to its quasi-molecular ion peak at *m*/*z* 165.0548 [M + H]^+^ and 147.0445 [M + H − H_2_O]^+^. In the ^1^H NMR spectrum, characteristic signals at *δ* 7.51 (1H, d, *J* = 6.0 Hz, H-2 and H-6), 7.47 (1H, s, H-8), 6.65 (1H, d, *J* = 9.0 Hz, H-3 and H-5), and 6.29 (1H, d, *J* = 15.0 Hz, H-8) indicated the presence of a *trans* double bond and para-substituent^33^. The ^13^C NMR spectrum gave rise to 9 carbon signals. Six carbon signals at *δ* 125.72 (C-1), 130.51 (C-2 and C-6), 116.19 (C-3 and C-5) and 160.03 (C-4) confirmed an aromatic group, and *δ* 168.35 was classified as one carbonyl group. In addition, the carbon signals at *δ* 144.59 and 115.79 supported the occurrence of *trans* double bond between C-7 and C-8. All the spectral data were in accordance with those of 4-hydroxycinnamic acid in the literature (Fig. 4)^34^.

### Anti-fungal activity validation

To validate the anti-fungal activity of compound **2**, the standard of 4-hydroxycinnamic acid was purchased from *ANPEL* Laboratory Technologies Inc. As shown in Table 2, the EC_50_ values of compound **2** and 4-hydroxycinnamic acid standard against *P. capsici* were 11.02 and 10.80 mg L^−1^. The results supported that 4-hydroxycinnamic acid was the main active compound of anti-fungal activity in moso bamboo extract.

**Table 2:** Anti-fungal activity of 4-hydroxycinnamic acid standard and compound **2**.

Concentration (mg L^−1^)	4-hydroxycinnamic acid standard				Compound 2			
	Inhibitory rate	Toxicity regression equation	EC_50_ (mg L^−1^)	95% CI (mg L^−1^)	Inhibitory rate	Toxicity regression equation	EC_50_ (mg L^−1^)	95% CI (mg L^−1^)
6.25	32.83 ± 0.35a	y = 3.9089x + 4.8249	10.80	8.77 ~ 12.28	29.87 ± 0.71b	y = 4.2640x + 4.7745	11.02	9.17 ~ 12.38
10.00	49.41 ± 0.76a				48.30 ± 1.05b			
12.50	59.30 ± 0.54a				58.77 ± 0.88b			
15.00	71.63 ± 0.82b				71.82 ± 0.52a			
20.00	81.72 ± 0.95b				83.43 ± 1.13a			

### Toxicological test of the 10% EW of BE

Based on the results of an anti-fungal test for phytopathogens in vitro and synergistic test, the 10% EW of BE was formulated according to standard methods (Table 3). Then, the acute toxicological test was performed. Test rats were given 5000 mg kg^−1^ b.wt. of 10% EW of BE, and no obvious symptoms of poisoning or death was observed. At the end of the observation period, the rats were dissected, and the main organs, such as the heart, lung, liver, spleen, stomach, intestine, and kidney, were observed to have no obvious abnormalities. The acute oral median lethal dose (LD_50_) value of 10% EW in male and female rats was greater than 5000 mg kg^−1^ b.wt. According to the acute oral toxicity grading standard^35,36^, the 10% EW of BE was a microtoxic substance. The acute percutaneous LD_50_ of 10% EW in male and female rats was greater than 5000 mg kg^−1^ b.wt. Additionally, this formulation was a microtoxic substance, according to the grading standard^37,38^.

**Table 3:** Formulation of the 10% EW of BE and 11.2% EW of BE · carbendazim.

Formulation	Composition	Content (%)
10% EW of BE	Bamboo leaf extract	10.0
	Solvent S	20.0
	Emulsifier E	4.0
	Emulsifier F	3.0
	Emulsifier G	3.0
	Anti-freeze agents N	4.0
	Thickeners M	0.2
	Water	55.8
11.2% EW of BE · carbendazim	Bamboo leaf extract:carbendazim (15:1)	11.2
	Solvent S	20.0
	Emulsifier J	4.0
	Emulsifier K	3.0
	Emulsifier L	3.0
	Anti-freeze agents Q	4.0
	Thickeners P	0.2
	Water	54.6

The results of the acute dermal irritation test showed that skin of both male and female rabbits was not stimulated or corroded, with the dose of 5000 mg kg^−1^ b.wt.

The stimulation response score and intensity results were statistically evaluated. The acute skin irritation score of the 10% EW of BE on rabbit was 0.0, the average index was 0.0, and no irritation was observed^39,40^. For the acute eye irritation of the BE formulation, the test rabbits were given 5000 mg kg^−1^ b.wt. The test results showed that the stimulation reaction did not disappear after 72 h (Table 4). The acute eye irritation integral index of the 10% EW of BE was 11.0, average index was 4.5 (less than 5 after 4 days). The index recovered to normal after 7 days, indicating moderate irritation^41–43^. After washing at 4 s, the average score was 1.5 (less than 5 after 48 h), and the score returned to normal at 72 h. A similar result was obtained in the test after washing at 30 s, which also indicated mild irritation.

**Table 4:** Evaluation of acute eye irritation about the 10% EW of BE.

No	Areas	Eyes without washing												Eyes were washed at 4 s								Eyes were washed at 30 s							
		Irritation response scores																											
		1 h		24 h		48 h		72 h		4 d		7 d		1 h		24 h		48 h		72 h		1 h		24 h		48 h		72 h	
		T	CK	T	CK	T	CK	T	CK	T	CK	T	CK	T	CK	T	CK	T	CK	T	CK	T	CK	T	CK	T	CK	T	CK
1	Conjunctiva	8	0	10	0	10	0	8	0	6	0	0	0	6	0	4	0	2	0	0	0	8	0	6	0	4	0	0	0
	Iris	0	0	0	0	5	0	5	0	0	0	0	0	0	0	0	0	0	0	0	0	0	0	0	0	0	0	0	0
	Cornea	0	0	0	0	0	0	0	0	0	0	0	0	0	0	0	0	0	0	0	0	0	0	0	0	0	0	0	0
	Score	8	0	10	0	15	0	13	0	6	0	0	0	6	0	4	0	2	0	0	0	8	0	6	0	4	0	0	0
2	Conjunctiva	6	0	10	0	8	0	6	0	4	0	0	0	8	0	4	0	2	0	0	0	8	0	6	0	2	0	0	0
	Iris	0	0	0	0	0	0	0	0	0	0	0	0	0	0	0	0	0	0	0	0	0	0	0	0	0	0	0	0
	Cornea	0	0	0	0	0	0	0	0	0	0	0	0	0	0	0	0	0	0	0	0	0	0	0	0	0	0	0	0
	Score	6	0	10	0	8	0	6	0	4	0	0	0	8	0	4	0	2	0	0	0	8	0	6	0	2	0	0	0
3	Conjunctiva	6	0	10	0	10	0	6	0	6	0	0	0	6	0	4	0	2	0	0	0	6	0	6	0	2	0	0	0
	Iris	0	0	0	0	5	0	0	0	0	0	0	0	0	0	0	0	0	0	0	0	0	0	0	0	0	0	0	0
	Cornea	0	0	0	0	0	0	0	0	0	0	0	0	0	0	0	0	0	0	0	0	0	0	0	0	0	0	0	0
	Score	6	0	10	0	15	0	6	0	6	0	0	0	6	0	4	0	2	0	0	0	6	0	6	0	2	0	0	0
4	Conjunctiva	6	0	6	0	6	0	6	0	2	0	0	0	6	0	4	0	2	0	0	0	8	0	6	0	4	0	0	0
	Iris	0	0	0	0	0	0	0	0	0	0	0	0	0	0	0	0	0	0	0	0	0	0	0	0	0	0	0	0
	Cornea	0	0	0	0	0	0	0	0	0	0	0	0	0	0	0	0	0	0	0	0	0	0	0	0	0	0	0	0
	Score	6	0	6	0	6	0	6	0	2	0	0	0	6	0	4	0	2	0	0	0	8	0	6	0	4	0	0	0
Total scores		26	0	36	0	44	0	31	0	18	0	0	0	26	0	16	0	6	0	0	0	30	0	24	0	12	0	0	0
Average irritation index		6.5		9.0		11.0		7.75		4.5		0.0		6.5		4.0		1.5		0.0		7.5		6.0		3.0		0.0	
Results		I.A.O.I. = 11.0; M.I.O.I. = 4.5; Mild and moderate irritation												I.A.O.I. = 6.5; M.I.O.I. = 1.5; Mild irritation								I.A.O.I. = 7.5; M.I.O.I. = 3.0; Mild irritation							

Note: T was treatment in this table; I.A.O.I. = Acute index of eye irritation; M.I.O.I. = The mean index of eye irritation.

### Field test

The 10% EW of BE and 11.2% EW of BE·carbendazim (Table 3) were used in a field containing *C. annuum* seedlings at the occurrence peak of pepper phytophthora blight. The results indicated that the control effect increased with application concentration, gradually (Table 5).

**Table 5:** Field test of 10% EW of BE control pepper phytophthora blight.

Formulation	Treatment (ml ha^−1^)	Disease index before spraying	Control effect ± SD (%)	
			3 days after spraying	7 days after spraying
10% EW of BE	3000	4.67	73.45 ± 2.30g	73.88 ± 1.10g
	6000	4.05	80.25 ± 1.60e	82.22 ± 0.85e
	9000	4.03	85.60 ± 2.15c	88.10 ± 0.88c
11.2% EW of BE · carbendazim	300	4.23	76.60 ± 2.43f.	80.61 ± 1.32f.
	600	4.25	80.94 ± 3.01d	82.82 ± 1.82d
	900	3.68	86.41 ± 0.90b	88.32 ± 1.75b
40% SC of carbendazim	900	3.99	89.22 ± 0.75a	91.98 ± 1.48a
CK	–	3.28	–	–

Different letters indicate significant differences according to Tukey HSD test comparisons (*p* < 0.05).

The control effect of 10% EW of BE was 85.60 ± 2.15%, with concentration of 9000 ml ha^−1^. The value of 40% SC of carbendazim was 89.22 ± 0.75% at the same investigation time. In the seventh day, the control effect of 11.2% EW of BE·carbendazim was 88.32 ± 1.75%, which showed a better control effect.

## Discussion and conclusion

These results demonstrated that the BE has better anti-fungal activity against phytopathogens *P. capsici*, *V. mali* and *F. graminearum*, which has the potential to be developed into a broad-spectrum fungicide. Anti-fungal activities of BE extracted from the moso bamboo leaves of different sources revealed that the activity was associated with location, light, temperature, humidity, soil environment, etc^44^. Some differences in the types and contents of secondary metabolites in bamboo leaves in different seasons and planting locations were reported^45,46^. These differences might be the reason that different BEs showed different activities in vitro.

In vitro studies on plants used in traditional medicine have been carried out in the field of microbiology, especially on phytopathogen growth, and some of these studies investigated the antimicrobial activity of *Allium sativum* L (garlic)^47^, *Psidium guajava* L. (guava)^48^, *Syzygium aromaticum* (L) Merrill & Perry^49^, and *Zingiber officinale* Roscoe (ginger)^50^. A paper about plants and antimicrobial activity with the objective of analyzing the past, present, and future suggested that the mechanisms of action, interactions with fungicides or with other plants and the pharmacokinetic profile of plant extracts are fundamental to research^51^. Research on synergism is very limited, and few studies have been reported^52,53^. Thus, in our research, we evaluated the synergism between extracts of moso bamboo leaf and carbendazim against *P. capsici* by using the hyphal growth rate method, and the results showed that the synergism of BE was significant.

In addition, 4-hydroxycinnamic acid was isolated and identified as the main anti-fungal compound in moso bamboo extract, which was validated with hyphal growth rate method^54^. In previous study, this compound was almost reported as an intermediate in the pharmaceutical and fragrance industry^55^, with anti-oxidant and anti-inflammatory properties^56,57^. The identification of the main active compound will promote the future use of moso bamboo resources and 4-hydroxycinnamic acid.

The optimized 10% EW of BE was stable and safe in accordance with national standards^58^. In this study, the results of the acute toxicological test indicated that the 10% EW of BE had no acute toxic effects, which provided an experimental basis for biosafety to develop and utilize it as a botanical fungicide^59^.

In this work, the 10% EW of BE and 11.2% EW of BE·carbendazim were identified to have a good control effect on pepper phytophthora blight. The extract of moso bamboo leaf has potential and broad application prospects for development as a new botanical fungicide formulation or a carbendazim compound preparation, especially to control pepper phytophthora blight. The standardized techniques for the application of moso bamboo leaf should also be taken into consideration to formulate commercial products.

## Materials and methods

### Extract of moso bamboo leaf

Dried moso bamboo leaves were collected from Ningguo County, Huoshan County, Shitai County, Qianshan County, Fanchang County, Shaodong County, Jianou County, and Chishui County, China. These voucher specimens of the samples collected from Ningguo County (designed No. 00-S2014-001 to No. 00-S2014-006 and No. 00-S2015-001), and the samples collected from other Counties (designed No. 00-S2014-007 to No. 00-S2014-013). All samples were deposited in School of Plant Protection, Anhui Agricultural University, Hefei 230036, China. The species was identified by Professor Yongde-Yue (State Forestry Administration Key Open Laboratory, International Centre for Bamboo and Rattan).

Bamboo leaf samples (100.00 kg) were extracted three times with 95% aqueous ethanol at room temperature (24 h × 20 L, total 72 L), referred the method established in this laboratory^60^. Then, a brown residue (2.65 kg) was yielded, which was the BE.

### Anti-fungal activity assays

The anti-fungal activity of BE was tested in vitro using the hyphal growth rate method of Arunyanark et al.^61^. The phytopathogens *F. graminearum*, *V. mali* Miyabe et Yamada, *P. capsici*, *B. dothidea*, *V. nashicola* and *B. cinerea* Pers were obtained from Plant Pathology Laboratory of Anhui Agricultural University. All of these phytopathogens inoculated at the centre of the plate and incubated in the dark at 28 °C. Subsequently, 0.5 g of filter-sterilized extracts dissolved in acetone (5 mL). The solution was added to potato dextrose agar medium (PDA medium) (95 mL), and then the medium was sub-packed in 6 sterilized petri dishes to obtaining drug-containing medium. Several fungal disks were made on the plate of phytopathogens with a hole punch with a 0.6 cm in diameter. The disk was picked up by an inoculation needle and placed lightly on drug-containing medium plates. This process was repeated 6 times per treatment, with reproducible results.

### Determination of the inhibitory rate

The plates were placed in the dark at 28 °C and photographed after 48 h. The calculation formula was as follows:

$$ Inhibitory\;rate\;(\% ) = \frac{Diameter\;of\;control - Diameter\;of\;treatment \, }{{Diameter\;of\;control - 0.6\; \, cm}} \times 100 $$

### Synergistic effect of BE

The BE extracted from the bamboo collected in Ningguo County was combined with carbendazim (96.0%) to evaluate the synergistic effect. The BE to carbendazim ratios were 5:1, 7:1, 9:1, 15:1, 49:1, and 99:1 according to mass.

The synergistic effect was expressed by the CTC. Firstly, the concentrations for 50% of maximal effect (EC_50_) value of BE and carbendazim were obtained from the previous results in this study and converted into the actual toxicity index (ATI). Then, the theoretical toxicity index (TTI) was assayed. A CTC value greater than 120 indicated synergism, a value between 80 and 120 indicated additive action, and less than 80 indicated antagonism. The calculation formula of CTC was as follows^62^:

$$ CTC = \frac{ATI \, }{{TTI}} \times 100 $$

### Analysis of main active component

To determine the main active ingredient in the extract of bamboo, the BE was sequentially extracted with petroleum ether, ethyl acetate and *n*-butyl alcohol. The extraction solutions were concentrated in *vacuo* to yield a petroleum ether-soluble fraction (321.40 g), an ethyl acetate-soluble fraction (508.25 g), and an *n*-butanol soluble fraction (412.10 g). Anti-fungal activity against *P. capsici* of these fractions was evaluated. Then, identification of active ingredients was assayed using PHPLC according to the results of activity tracking.

The active compound was analyzed by ultraviolet chromatography (UV), nuclear magnetic resonance (NMR) and high resolution electrospray ionization mass spectra (HRESIMS). Then, the anti-fungal activity of this compound was validated and compared with standard (purity ≥ 98.00%), which purchased from *ANPEL* Laboratory Technologies Inc. (Shanghai, China).

### BE formulation

Stable 10% EW of BE was formulated according to national standards. The formula was optimized by investigating solvents, emulsifiers and other auxiliaries. Acute toxicity tests were performed according to national standards, including acute oral toxicity, acute percutaneous toxicity, acute dermal irritation and acute eye irritation.

The 10% EW of BE was dosed to a desired drug concentration using soybean oil as a solvent, with concentration of 5000 mg kg^−1^ b.wt. Ten male rats and 10 female rats were used to evaluate the acute oral toxicity, and the results of poisoning symptoms and death were recorded after 14 days of one oral exposure. A similar method was used to assess acute percutaneous toxicity, and the touching method was one percutaneous exposure.

Four rabbits (2 male and 2 female) with healthy skin were chosen for the acute dermal irritation test. The hair of the rabbits (6 cm^2^) was removed from the back before the test, and 0.1 mL of formulation was used. Then, the medication section was covered with gauze after application, and normal skin was used as a blank control. Similarly, healthy rabbits with good eyes were used to evaluate the acute eye irritation of BE formulation. The lower eyelid of one side was gently pulled down, and 0.1 mL of the formulation was added to the conjunctival sac. To prevent the liquid from overflowing, the eyelid was closed for approximately 1 min and washing was avoided for 24 h after the application. The contralateral eye was the blank control. The results were recorded at 1, 24, 48 and 72 h after application. If there was no stimulatory reaction at 72 h, the test was terminated. If a reaction appeared, the damage and reversibility continued to be observed for up to 21 days. If the response to stimulation did not resolve in 72 h, another 4 rabbits were selected to observe the eye wash effect, after instilling the formulation, the eyes were washed with physiological saline for 5 min at 4 s and 30 s, and the reaction was observed. Additional 8 rabbits were observed for eye reaction, eyes were washed at 4 s and 30 s after applying formulation.

### Field test

The test was conducted in a greenhouse in Lujiang County, Anhui Province. *C. annuum*. was chosen as the test subject for the field test, and the disease was pepper phytophthora blight. The test plot soil was loam, and the management level of the greenhouse was consistent with others. Fertilization, irrigation and drainage were not performed during the test, which satisfied the requirements of the test^63^.

40% SC of carbendazim was chosen as positive controls, and the concentration was 900 mL ha^−1^. The 10% EW of BE was assayed at three concentrations, 3000, 6000, and 9000 mL ha^−1^, and the 11.2% compound EW of BE·carbendazim was tested at 300, 600, and 900 mL ha^−1^. In addition, the blank control was sprayed water. Each treatment repeated three times. The area of each plot was 600 m^2^, these plots were arranged randomly, with protected rows and areas around the test field.

A 3WBD-16C electric sprayer was used to spray the formulation evenly on the leaves, and the amount of spray liquid was 750 L ha^−1^. Five points per plot were chosen before spraying, and 200 strains were fixed at each point. The test was assayed at the occurrence peak of pepper phytophthora blight*.* Before the application of these formulations, the disease index was investigated, and the level of disease on the marked leaves of each plot was investigated on the third and seventh days after spraying. Then, the disease index and treatment effects were calculated.

### Statistical analysis

Data are expressed as mean ± standard deviation (SD). Means were compared using Tukey HSD test, the differences in the mean values of *P* < 0.05 between treatment groups were set as the significance threshold. Data from field tests were analyzed with analysis of variance (ANOVA), significant ANOVAs were then followed by mean separation with a Tukey HSD. The test data obtained from each dose response bioassay were subjected to probit analysis, and EC_50_ values and their 95% confidence intervals were estimated. Comparisons of the EC_50_ values were based on the overlap of the confidence intervals. Analyses were conducted using the statistical package SPSS 14.0.

### Ethics statement

The animal testing procedures have been approved by the Code of Ethics and reviewed and implemented according to the guidelines of the Animal Care and Use Committee of Anhui Medical University (number: LLSC20150348). All the procedures were carried out in full accordance with the Helsinki Declaration on Animal Rights. Adequate care was taken to minimize pain and discomfort. Animals were given food and water ad libitum. All protocols in this study were carried out in compliance with the ARRIVE guidelines.

Forty rats were used: male rats (n = 20) and female rats (n = 20) both were BALB/c OlaHsd obtained from Anhui Medical University, China. At the time of these tests the rat had body weights of 200 ± 3 g (mean ± SEM). Eight rabbits were used: male rats (n = 4) and female rats (n = 4) both were New Zealand white rabbits, obtained from Anhui Medical University, China. At the time of these tests the rat had body weights of 2500 ± 50 g (mean ± SEM).

## Supplementary Information

Supplementary Figures.
