# Supplementary material for: Anti-fungal activity of moso bamboo (Phyllostachys pubescens) leaf extract and its development into a botanical fungicide to control pepper phytophthora blight
Source: Sci Rep. 2021 Feb 18;11:4146. doi: 10.1038/s41598-021-83598-y (PMC7892876; doi:10.1038/s41598-021-83598-y)
Supplement: Supplementary file 1 — Supplementary Figures. [file 41598_2021_83598_MOESM1_ESM.pdf]

Anti-fungal activity of moso bamboo (*Phyllostachys pubescens*) leaf extract and its development into a botanical fungicide to control pepper phytophthora blight

Min Liao[1,2], Xuexiang Ren[3], Quan Gao[1,2], Niuniu Liu[4], Feng Tang[5], Ge Wang[6] & Haiqun Cao[1,2,*]

1 Anhui Province Key Laboratory of Crop Integrated Pest Management, School of Plant Protection, Anhui Agricultural University, Hefei, China

2 Anhui Province Engineering Laboratory for Green Pesticide Development and Application, School of Plant Protection, Anhui Agricultural University, Hefei, China

3 Institute of Plant Protection and Agro-products Safety, Anhui Academy of Agricultural Sciences, Hefei, China

4 School of Resource and Environment, Anhui Agricultural University, Hefei, China

5 State Forestry Administration Key Open Laboratory, International Centre for Bamboo and Rattan, Beijing, China

6 Key Laboratory of Bamboo and Rattan Science and Technology of the State Forestry Administration, Department of Bio-materials, International Centre for Bamboo and Rattan, Beijing, China

* caohq@ahau.edu.cn

Supplementary information

Fig. S1 ^{1}H-NMR for compound **2** (300 MHz, DMSO, δ ppm)

Fig. S2 ^{13}C-NMR for compound **2** (75 MHz, DMSO, δ ppm)

Fig. S3 Dept 135° for compound **2** (300 MHz, DMSO, δ ppm)

Fig. S4 HMBC for compound **2** (300 MHz, DMSO, δ ppm)

Fig. S5 HSQC for compound **2** (300 MHz, DMSO, δ ppm)

Fig. S6 HRESIMS for compound **2**

Fig. S7 UV spectral data for compound **2**

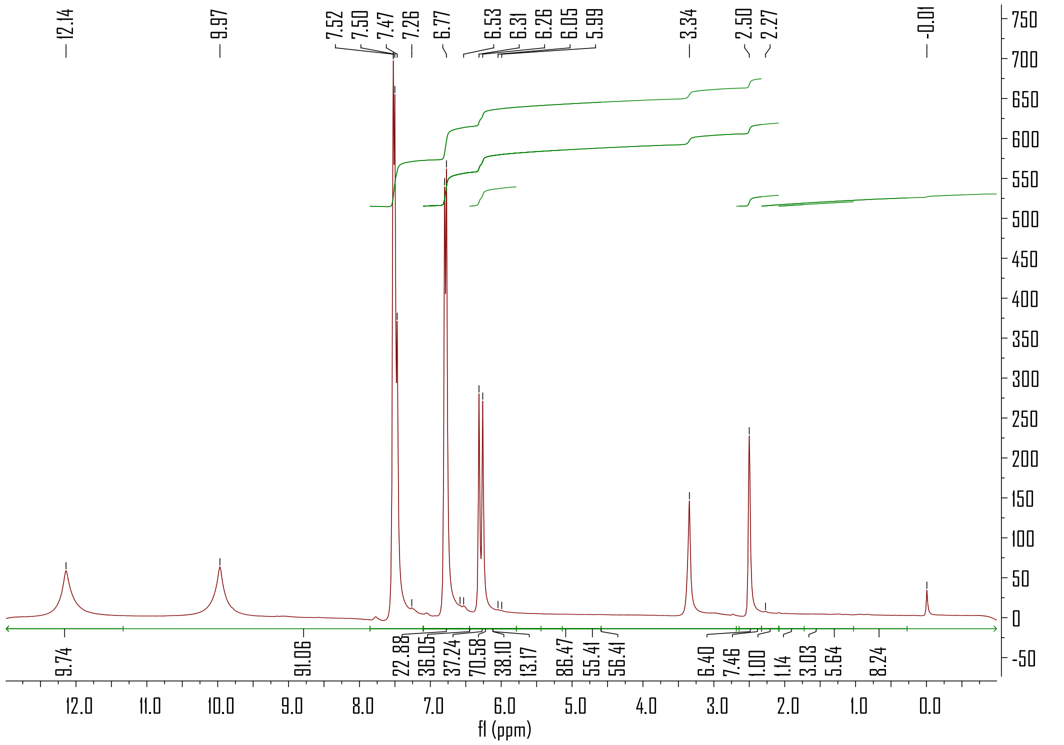

Fig. S1 ^{1}H-NMR for compound **2** (300 MHz, DMSO, δ ppm)

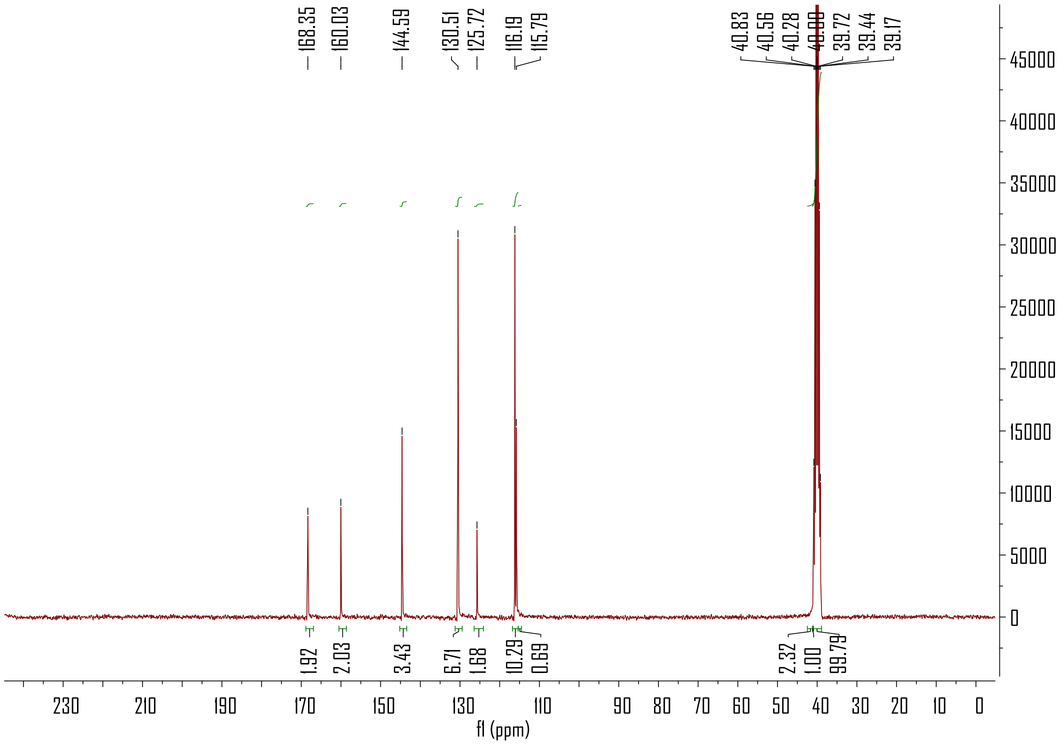

Fig. S2 ^{13}C-NMR for compound **2** (75 MHz, DMSO, δ ppm)

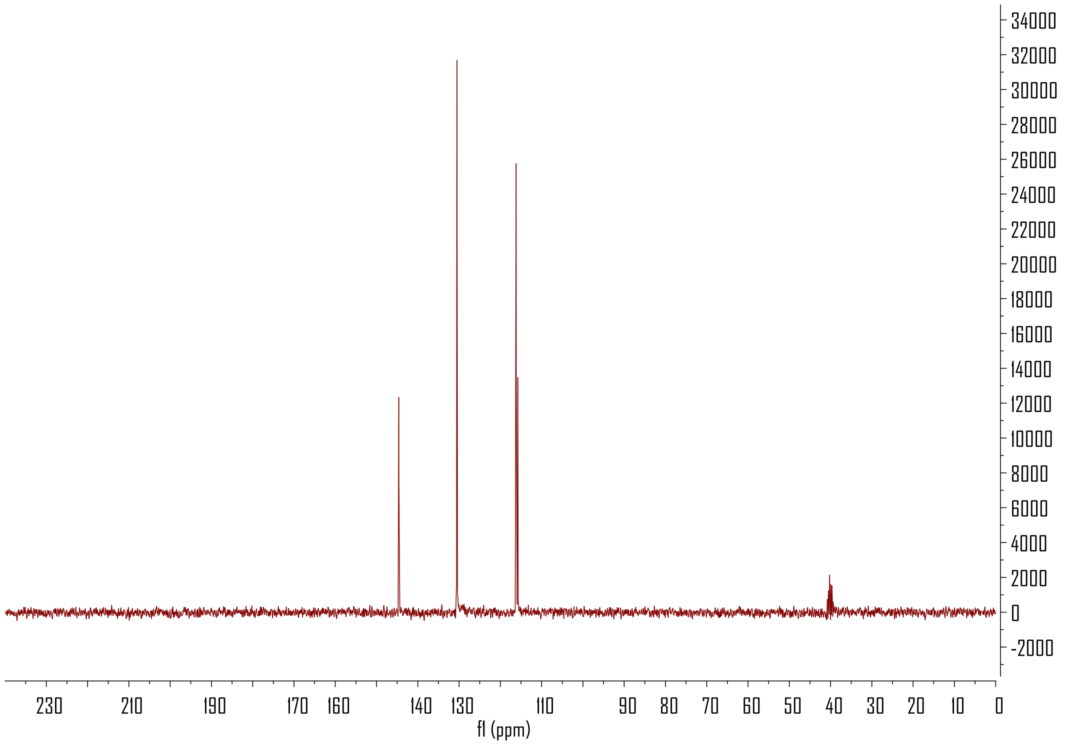

Fig. S3 Dept 135° for compound **2** (300 MHz, DMSO, δ ppm)

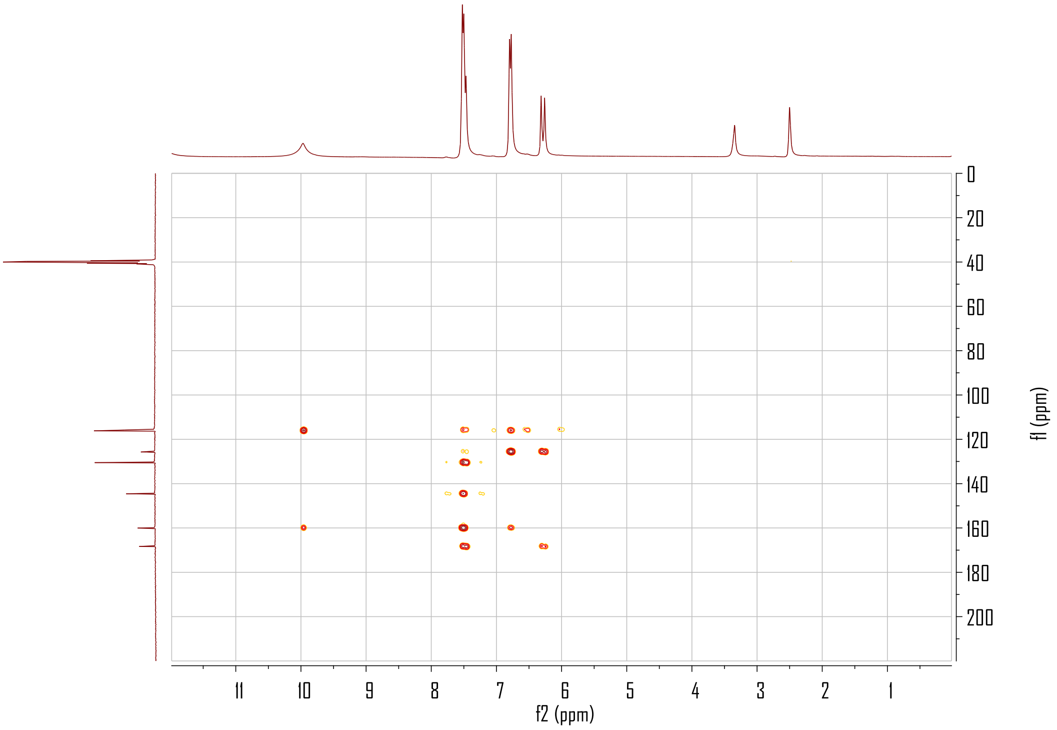

Fig. S4 HMBC for compound **2** (300 MHz, DMSO, δ ppm)

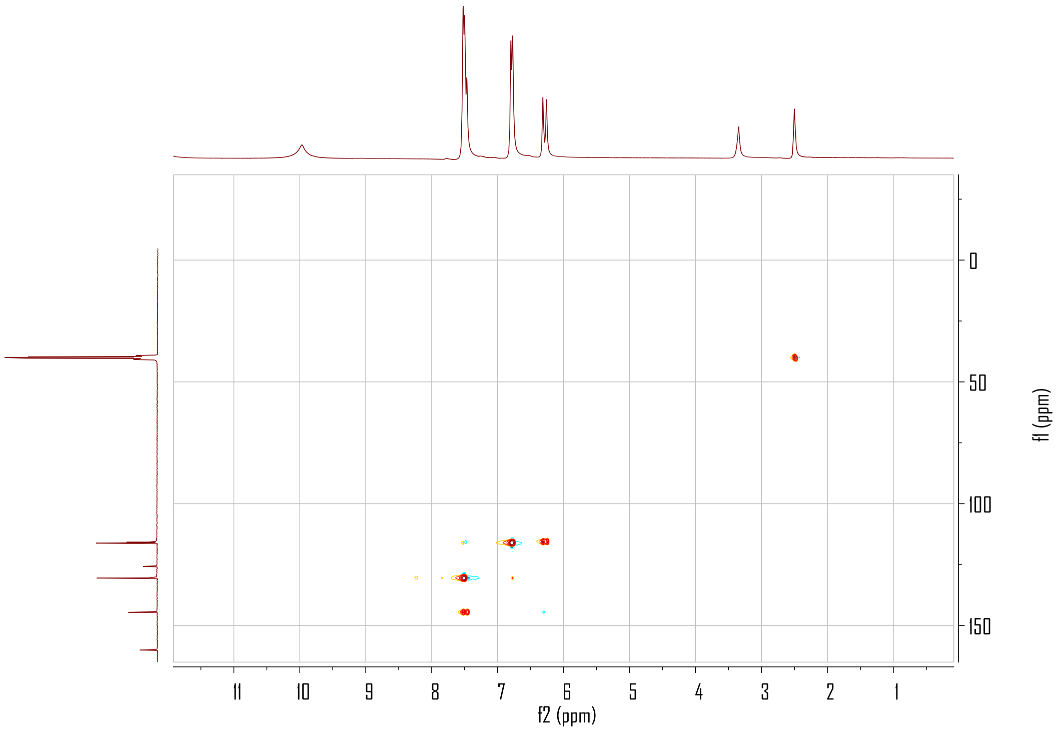

Fig. S5 HSQC for compound **2** (300 MHz, DMSO, δ ppm)

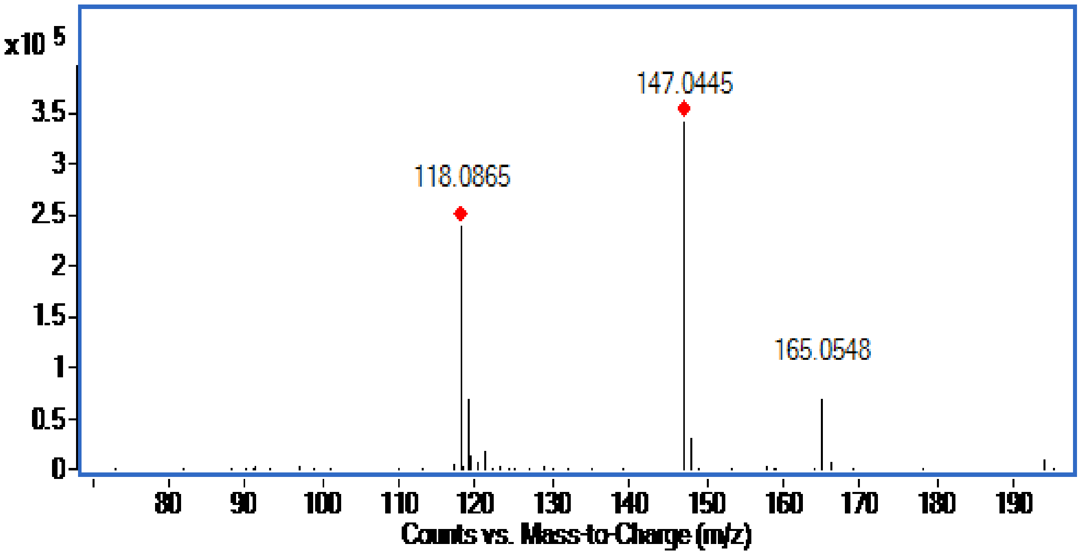

Fig. S6 HRESIMS for compound **2**

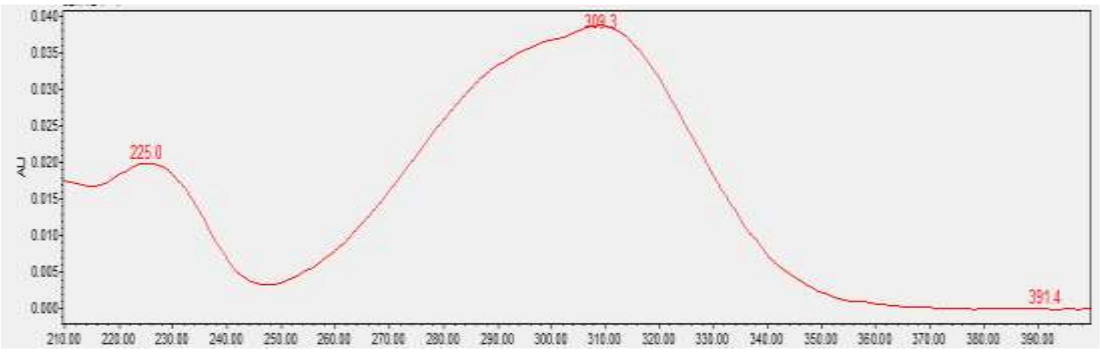

Fig. S7 UV spectral data for compound **2**
